# Lytic viral replication and immunopathology in a cytomegalovirus-induced mouse model of secondary hemophagocytic lymphohistiocytosis

**DOI:** 10.1186/s12985-017-0908-0

**Published:** 2017-12-19

**Authors:** Ellen Brisse, Maya Imbrechts, Tania Mitera, Jessica Vandenhaute, Carine H. Wouters, Robert Snoeck, Graciela Andrei, Patrick Matthys

**Affiliations:** 1 Laboratory of Immunobiology, Rega Institute, Department of Microbiology and Immunology, KU Leuven, Leuven, Belgium; 2 Laboratory of Pediatric Immunology, Department of Microbiology and Immunology, University Hospital Gasthuisberg, KU Leuven, Leuven, Belgium; 3 Laboratory of Virology and Chemotherapy, Rega Institute, Department of Microbiology and Immunology, KU Leuven, Leuven, Belgium

**Keywords:** Hemophagocytic lymphohistiocytosis, HLH, macrophage activation syndrome, MAS, mouse model, mouse cytomegalovirus, MCMV, immunopathology, virus-mediated pathology

## Abstract

**Background:**

Hemophagocytic lymphohistiocytosis (HLH) is a rare immunological disorder caused by unbridled activation of T cells and macrophages, culminating in a life-threatening cytokine storm. A genetic and acquired subtype are distinguished, termed primary and secondary HLH, respectively. Clinical manifestations of both forms are frequently preceded by a viral infection, predominantly with herpesviruses. The exact role of the viral infection in the development of the hemophagocytic syndrome remains to be further elucidated.

**Methods:**

We utilized a recently developed murine model of cytomegalovirus-associated secondary HLH and dissected the respective contributions of lytic viral replication and immunopathology in its pathogenesis.

**Results:**

HLH-like disease only developed in cytomegalovirus-susceptible mouse strains unable to clear the virus, but the severity of symptoms was not correlated to the infectious viral titer. Lytic viral replication and sustained viremia played an essential part in the pathogenesis since abortive viral infection was insufficient to induce a full-blown HLH-like syndrome. Nonetheless, a limited set of symptoms, in particular anemia, thrombocytopenia and elevated levels of soluble CD25, appeared less dependent of the viral replication but rather mediated by the host’s immune response, as corroborated by immunosuppressive treatment of infected mice with dexamethasone.

**Conclusion:**

Both virus-mediated pathology and immunopathology cooperate in the pathogenesis of full-blown virus-associated secondary HLH and are closely entangled. A certain level of viremia appears necessary to elicit the characteristic HLH-like symptoms in the model.

## Background

Hemophagocytic lymphohistiocytosis (HLH) is a severe and potentially lethal hyperinflammatory syndrome, characterized by persistent high fever, hepatosplenomegaly, pancytopenia, coagulopathy, hyperferritinemia, hemophagocytosis and a cytokine storm. A primary, inherited form and a secondary, acquired form can be distinguished [1]. Secondary HLH was first described in adults, in association with active viral infections, upon which it was termed a “reactive, virus-associated hemophagocytic syndrome” [2]. Nowadays, the spectrum of secondary HLH has expanded to include associations with bacterial, fungal and parasitic infections, underlying malignancies and autoimmune or autoinflammatory diseases. Virus-associated secondary HLH constitutes the largest group of secondary HLH, accounting for approximately 35% of patients [3]. Noteworthy, viruses are also frequently implicated in malignancy-associated secondary HLH, either as a co-trigger of the HLH episode or as the cause of the underlying malignancy [4]. Furthermore, clinical manifestations of primary HLH are predominantly precipitated by a viral infection [5]. Several viral agents are capable of triggering HLH, including parvovirus B19, influenza virus, enteroviruses and adenoviruses, but herpesviruses like Epstein-Barr virus (EBV) and human cytomegalovirus (HCMV) are the most common [3, 6]. Intriguingly, most of the described triggering viruses have a lytic replication cycle, which incites a direct cytopathic effect on the infected cell, inducing host tissue damage. Tissue damage in turn will result in the release of multiple ‘alarmins’ or danger-associated molecular patterns (DAMPs), molecules that were described to contribute directly to disease pathogenesis in HLH, in addition to eliciting immunopathology [7]. Thus, two pathogenic processes may synergize in virus-associated HLH: immune-mediated pathology and direct, virus-mediated cytolytic effects.

Current pathophysiological insights into the role of viruses in HLH have mostly been derived from animal models of primary HLH, in which mutant mouse strains are inoculated with lymphocytic choriomeningitis virus (LCMV) [8–11]. However, unlike the most abundant viral triggers of HLH, this Arenavirus carries a (-)ssRNA genome and is known to be noncytopathic *in vivo* [12]. Hence, LCMV will conceivably stimulate other pathogen receptors and mediate different immune responses than herpesviruses would, resulting in a course of infection that may not fully reflect the underlying mechanisms of most virus-induced HLH cases. Therefore, to more specifically study the pathogenic mechanisms exerted by DNA viruses in secondary HLH, we recently developed a new mouse model of herpesvirus-induced secondary HLH, using mouse cytomegalovirus (MCMV) infection in susceptible wild-type (WT) and IFN-γ-deficient BALB/c mice [13]. Like EBV and HCMV, MCMV has a lytic replication cycle, mediating direct cytopathic effects in infected tissues and making this mouse model an interesting tool to investigate the respective roles of uncontrolled viral replication and immunopathology in HLH pathogenesis. In the current study, the contribution of lytic viral replication to disease development was explored using different strategies. Organ titers of infectious MCMV were correlated to the severity of key HLH symptoms in the model. The possible pathogenic effects of a sustained, non-cytolytic viral infection were imitated using different methods. Firstly, the effect of ongoing, non-specific immune receptor triggering was studied by repeatedly administering CpG and/or polyinosinic-polycytidylic acid (Poly(I:C)) as continuous Toll-like receptor (TLR) triggers. Secondly, to mimic MCMV-specific immune cell triggering in a more precise way, UV-inactivated, replication-deficient MCMV particles were repeatedly administered. Lastly, the lytic viral replication of MCMV was inhibited *in vivo* using either polyclonal antibody neutralization or pharmacological inhibition by antiviral cidofovir treatment. To decipher the role of immunopathology, a treatment with immunosuppressive dexamethasone was tested in the MCMV-induced mouse model.

## Methods

### Experimental design of the MCMV-induced secondary HLH mouse model

**Fig. 1:**
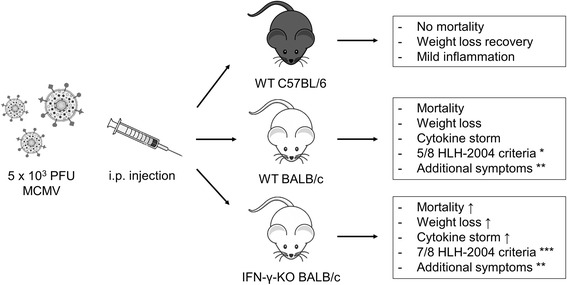
Schematic representation of the murine model of virus-associated secondary HLH. HLH-like disease is present in WT and IFN-γ-KO BALB/c mice, not in WT C57BL/6 mice. Mice are infected i.p. with 5 x 10^3^ PFU of MCMV and analyzed 5 days post infection. * = fever, pancytopenia, hemophagocytosis, hyperferritinemia and elevated soluble CD25 levels; ** = lymphadenopathy, liver dysfunction and decreased NK cell numbers; *** = in addition to WT BALB/c mice also splenomegaly, coagulopathy and decreased NK cell cytotoxicity. ↑ = more pronounced as compared to infected WT BALB/c mice; NK = natural killer; KO = knockout; MCMV = mouse cytomegalovirus; PFU = plaque-forming units; WT = wild-type

IFN-γ-knockout (KO) mice on BALB/c background, corresponding WT BALB/c mice and WT C57BL/6 mice were bred under specific pathogen-free conditions in the Experimental Animal Centre of KU Leuven. Mice of 4-6 weeks old were age- and sex-matched within each experiment. Experiments were carried out in a conventional animal facility in accordance with the recommendations of the Animal Ethics Committee of KU Leuven. The protocol was approved by the Animal Ethics Committee of KU Leuven (P055/2012). Per mouse, an inoculum of 5 x 10^3^ plaque-forming units (PFU) MCMV (Smith strain, VR-1399, ATCC) in 100 μl PBS was injected intraperitoneally (i.p.) on day 0. Stocks of MCMV were prepared from homogenates of salivary glands of NMRI mice after 2-3 weeks infection with a sublethal dose of MCMV, as a 10% w/v solution in MEM medium. Only the clear supernatant of salivary glands homogenates was used (600g, 10min, 4°C) [14]. PBS-injected mice were included as controls. Weight and rectal temperature of the mice was measured daily. Mice were euthanized with Nembutal (Ceva) on day 2 post infection (p.i.), at the first signs of inflammation, or on day 5 p.i., according to institutional ethical policies, when chronic weight loss exceeded 20-25% of initial body weight or when body temperature dropped below 34.5 °C. The resulting murine HLH model, as described in reference [13] is illustrated in Fig. 1. Unless specified otherwise, immunocompetent WT BALB/c mice were used in the *in vivo* experiments.

### Quantification of viral titers, polyclonal neutralization and UV-inactivation of MCMV

The amount of infectious virus present in different organs was determined by a plaque assay using a tenfold titration of the supernatant of organ lysates on a monolayer of C127I cells (CRL-1616, ATCC). Briefly, each organ sample was thawed, weighed and lysed in 1 ml of MEM medium with 2% heat-inactivated fetal bovine serum, 1% non-essential amino acids, 1% sodium pyruvate, 1% penicillin/streptomycin/glutamine and 1M HEPES. Only clear supernatant was used (10 min, 600g, 4°C), debris was discarded. Samples were serially diluted in a 96-well plate, using 100 μl per well, in quintuplicate. Organ lysates were incubated on the C127I monolayer for 2 hours (37°C, 5% CO_2_). After removal of the lysates and addition of MEM medium (components listed above), the cell cultures were further incubated for 7 days, after which plaques were counted using light microscopy. Detection limit of the assay was 2 PFU per organ. To inactivate the virus, two approaches were used, UV-inactivation and neutralization of MCMV using polyclonal antibodies. For UV inactivation, a stock solution of MCMV was incubated for 10 minutes under continuous agitation at a distance of 5 cm underneath UV light (optimal conditions were established in a pilot experiment, determining minimal exposure required for complete inactivation). Mice were injected i.p. with 5 x 10^3^ or 10^4^ PFU of UV-inactivated MCMV at day 0, 2 and 4. Analysis was at day 5. UV-inactivated virus was freshly prepared each time point and complete inactivation was confirmed via plaque assay. For MCMV neutralization, polyclonal anti-MCMV antibodies (IgG, neutralizing titer 64) were kindly provided by Prof. Dr. H. J. Nauwynck (Ghent University). The isolation and titer determination of these antibodies have previously been described [15]. Diluted MCMV stock solution was incubated for 1h at 37°C with a 1/32 dilution of neutralizing antibodies, prior to infection of the mice on day 0. As an isotype control, polyclonal IgG antibodies were purified from serum of naïve BALB/c mice, and similarly incubated with diluted MCMV stock solution.

### Administration of CpG, Poly(I:C), cidofovir or dexamethasone

CpG (50 or 100 μg per mouse, ODN1826, Integrated DNA Technologies) and/or Poly(I:C) (50 or 200 μg per mouse, Sigma-Aldrich) were administered i.p. to naive mice on day 0, 2, 4, 6 and 8. Analysis was on day 9. A single shot of cidofovir (50mg/kg body weight, kindly provided by Gilead Sciences) was injected i.p. either at 2 hours or at 2 days p.i. with MCMV to limit viral replication. A control group of naive mice was identically treated with cidofovir to rule out drug-specific effects. Dexamethasone 21-phosphate disodium salt (DEX, Sigma-Aldrich) was injected i.p., daily, starting on day 2 p.i. with MCMV, in a dose-response set-up, comparing 1, 2, 4, 8 and 16 mg/kg body weight. As these different concentrations did not result in clinical or laboratory differences, the data were combined in one DEX-treated experimental group. All molecules were diluted to the required concentration in sterile PBS. Control MCMV-infected mice were injected daily with an equal volume of PBS. All experiments were performed using a randomized design to avoid cage effects.

### Blood analysis and quantification of liver enzymes

Blood samples were obtained via cardiac puncture with heparin (LEO Pharma). Blood cell analysis was performed with a Cell-Dyn 3700 Hematology Analyzer (Abbott Diagnostics). Plasma concentrations of alanine transaminase (ALT) were measured spectrophotometrically using a UV-kinetic method according to the manufacturer’s instructions (ALT (SGPT) Reagent Set, Teco Diagnostics).

### Quantification of soluble CD25 (sCD25) and ferritin using ELISA

The protein level of sCD25 was determined in plasma according to the manufacturers’ instructions (DuoSet, R&D Systems). The lower ELISA detection limit was 78 pg/ml. A sandwich ELISA detecting the ferritin heavy chain was kindly offered by Dr. Paolo Santambrogio (San Raffaele Scientific Institute, Il Dipartimento di Biotecnologie, Milan, Italy) [16]. The lower ELISA detection limit was 9.76 ng/ml.

### Single-cell suspensions, cytospins and flow cytometry

For single-cell suspensions, white blood cells were obtained from blood after lysis of red blood cells with NH_4_Cl. Lung white blood cells were obtained from density gradient centrifuged lung cell suspensions (Percoll 40% and 72%, GE Healthcare). Lymph node cells were extracted from both inguinal lymph nodes. For cytospin preparations, single-cell suspensions were spun on a glass slide and stained with H&E. For flow cytometry, cell suspensions were incubated with anti-CD16/anti-CD32 (Miltenyi Biotec) and stained with the following monoclonal antibodies: CD3e (clone 145-2C11), CD8a (53-6.7), CD25 (PC61.5), CD49b (DX5), CD69 (H1.2F3), CD122 (5H4) (eBioscience, BD Biosciences or BioLegend). Dead cells were excluded using propidium iodide (PI) or Zombie Aqua Fixable Viability Dye (BioLegend). Samples were run with a FACSCalibur using CELLQuest software or an LSR Fortessa X-20 using FACSDiva software (all BD Biosciences). Live singlet cells (PI^-^ or ZombieAqua^-^) were analyzed with FlowJo (Version 10).

### Statistical analysis

Data with two experimental groups were analyzed via a two-tailed nonparametric Mann-Whitney U test. For comparison of three or more groups, a nonparametric Kruskal-Wallis test was performed, followed by Dunn’s multiple comparison post-test. For correlation analyses, a two-tailed nonparametric Spearman test was applied. Outliers were identified using a Grubbs’ test and all deviated more than 3 times the standard deviation from the group median. GraphPad Prism 5.00 was utilized.

## Results

### Infectious viral titers are higher in MCMV-susceptible mice, but do not correlate with severity of HLH-like symptoms

**Fig. 2:**
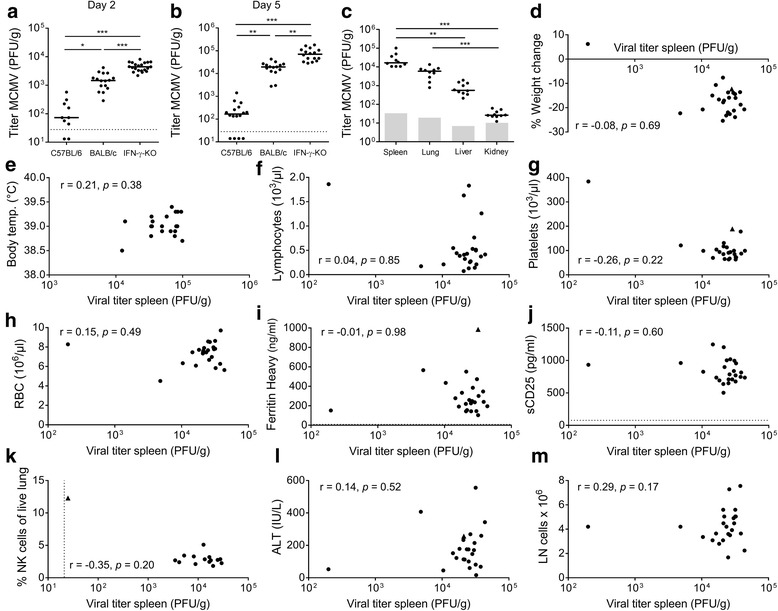
Spleen viral titers are not correlated with the severity of HLH-like symptoms. **a** Comparison of spleen viral titer (PFU/g) in different mouse strains on day 2 post infection (p.i.) and **b** day 5 p.i.. **c** Titer of infectious virus in spleen, lung, liver and kidney (PFU per g organ tissue) of WT BALB/c mice on day 5 p.i.. **d-m** Correlation analysis between the infectious viral titer in spleen (PFU/g) on day 5 p.i. and **d** percentage change in body weight relative to body weight at day 0 p.i., **e** rectal body temperature (°C) on day 2 p.i., **f-h** absolute lymphocyte, platelet and red blood cell (RBC) count in whole blood, plasma concentration of **i** ferritin heavy chain (ng/ml) and **j** sCD25 (pg/ml), **k** percentage of NK cells in lungs, gated as CD122^+^CD49b^+^CD3^-^ZombieAqua^-^ cells, **l** plasma concentration of ALT (IU/L) and **m** total cell number of inguinal lymph nodes. **a-m** Dots represent individual animals. Outliers are depicted with a triangle ▲. Horizontal bars refer to median group values. **d, f-m** Data obtained on day 5 p.i.. **a-c** Depicted data are from 1-3 experiments and representative of >5 or **d-m** 8 independent experiments with >5 mice per experimental group. **d, f-j, l-m** Data from 1 experiment with 25 BALB/c MCMV mice. **e, k** Data from 1 experiment with 20 and 15 BALB/c MCMV mice, respectively. **a-m** Detection limit of the plaque assay is 2 PFU/organ, represented by grey boxes or a dotted line. **i, j** Lower ELISA detection limits are indicated with a dotted line. ALT = alanine transaminase, KO = knockout, LN = lymph node, NK = natural killer, PFU = plaque-forming units, r = Spearman correlation coefficient, sCD25 = soluble CD25, temp. = temperature, WT = wild-type. *p* > 0.05; ** *p* < 0.01; *** *p* < 0.001; Kruskal-Wallis with Dunn’s post-test for multiple comparisons, Spearman test for correlation analysis

C57BL/6 mice and BALB/c mice are known to be respectively resistant and susceptible to mouse cytomegalovirus (MCMV) infection [17, 18]. When wild-type (WT) C57BL/6, WT BALB/c and IFN-γ-KO BALB/c mice were infected with MCMV, the infectious viral titer in these strains differed significantly from day 2 up to day 5 post infection (Fig. 2a, b). The viral load was generally higher in IFN-γ-KO mice than in corresponding WT BALB/c mice, while WT C57BL/6 mice displayed lower to undetectable viral titers as they managed to rapidly control the virus. As infected IFN-γ-KO mice show a more complete HLH spectrum when compared to infected WT BALB/c mice, and as infected WT C57BL/6 mice display little to no HLH-like symptoms [13], this raised the question whether inordinate replication of MCMV, a cytolytic virus, could be the main cause of the observed HLH-like syndrome.

To assess a possible relationship between the development of the HLH-like syndrome and the rate of viral replication, with its concomitant cytolytic effects, a correlation analysis was performed between the viral load in WT BALB/c mice and the severity of different HLH symptoms. Day 5 post infection was chosen for this analysis since the highest viral titers were observed at this time point (Fig. 2a, b). The spleen viral titer was selected since, at this time point in the model, MCMV is predominantly present in the spleen, compared to lung, liver and kidneys (Fig. 2c). Of note, an even higher viral load was often detected in salivary glands, in line with reports identifying the acinar glandular epithelial cells as the main site of ongoing productive infection. However, since the virus may persist for weeks in the glands even when viremia has been successfully cleared and MCMV is no longer detectable in any visceral organs, salivary gland titers do not reflect the systemic disease course as accurately as spleen viral titers do [18–21]. Key symptoms developing in MCMV-infected BALB/c mice were previously described [13] and include severe weight loss, fever on day 2 post infection, hypothermia on day 5 post infection, pancytopenia, hemophagocytosis in different organs and in peripheral blood, hyperferritinemia, elevated plasma levels of soluble CD25 (sCD25), a decreased percentage of natural killer (NK) cells in different organs and in peripheral blood, increased plasma levels of liver enzymes (alanine transaminase, ALT), and enlarged lymph nodes. A Spearman correlation test was performed on 8 independent experiments, with 15 to 25 infected mice in each experiment. In none of these experiments could a significant relationship be detected between spleen viral titers of MCMV and the extent of body weight loss post infection, fever, lymphopenia, thrombocytopenia, anemia, hyperferritinemia, plasma levels of sCD25, remaining percentage of NK cells in lung, plasma levels of ALT, or the extent of lymphadenopathy (Fig. 2d-m). As some outliers were present in the dataset, depicted as triangles in Fig. 2, the correlation analyses were additionally ran in the absence of outliers, however once more, no significant correlations were detected. Thus, although the infectious viral titer is the highest in mouse strains developing an HLH-like syndrome post MCMV infection, the titers do not correlate with the severity of key HLH features.

### Persistent stimulation of TLR3, in contrast to TLR9, is not sufficient to induce an HLH-like syndrome in WT BALB/c mice

**Fig. 3:**
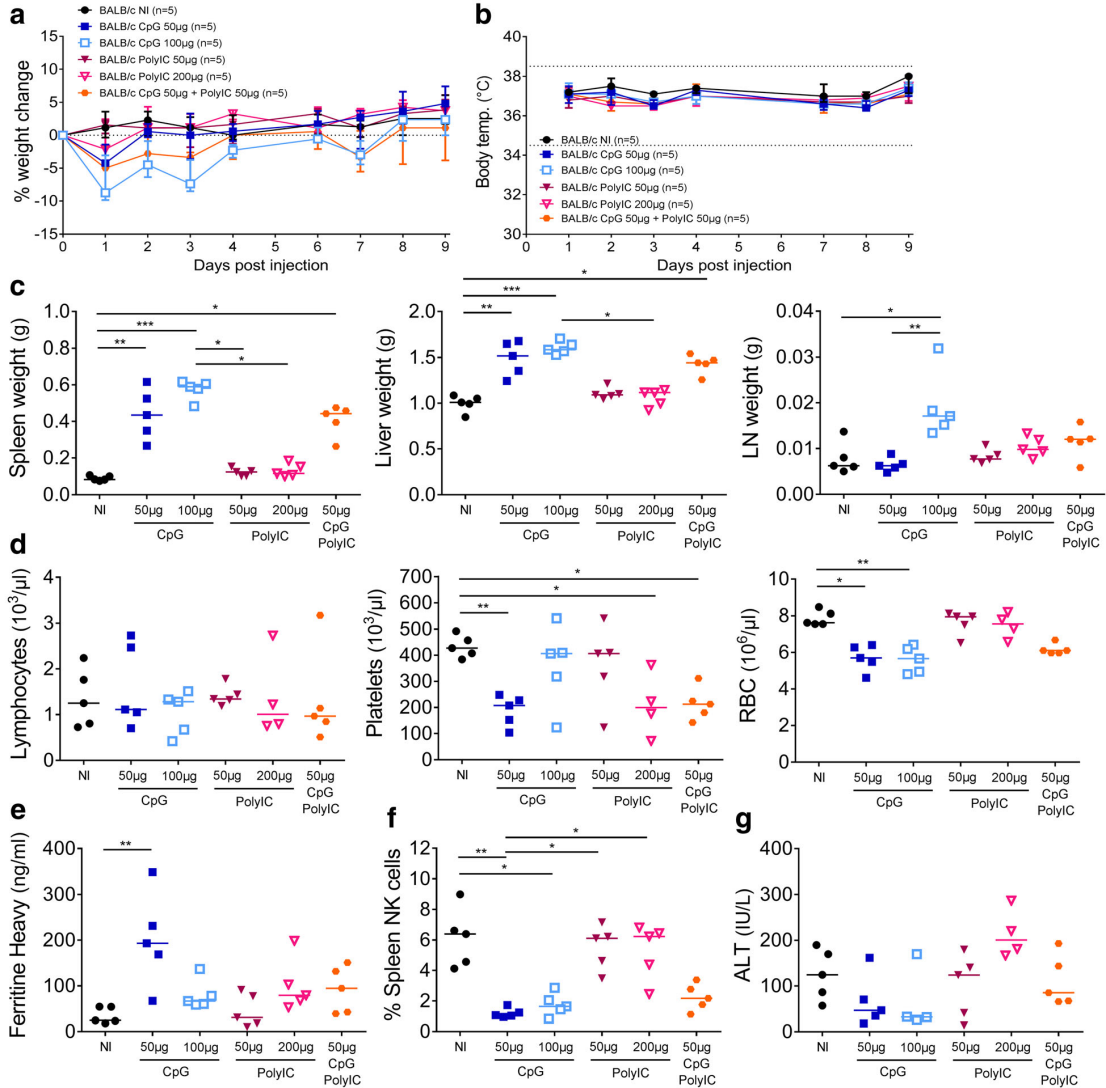
Repeated CpG, but not Poly(I:C) injections induce a mild HLH-like syndrome in WT BALB/c mice. **a** Percentage change in body weight relative to initial body weight at day 0. Median with interquartile range of 5 mice per experimental group. **b** Rectal body temperature (°C). Dotted line = 38.5°C (fever) or 34.5°C (endpoint as an indication of mortality). Median with interquartile range of 5 mice per experimental group. **c** Absolute spleen, liver and lymph node (LN) weight (g). Two inguinal LN were measured. **d** Absolute lymphocyte, platelet and red blood cell (RBC) count in whole blood. **e** Plasma concentration of the ferritin heavy chain (ng/ml). **f** Percentage of NK cells in spleen, analyzed as CD49b^+^CD122^+^ of CD3^-^PI^-^ splenocytes. **g** Plasma concentration of ALT (IU/L). **c, d, f, g** Dots represent individual animals. **e** Dots represent the average of two dilutions for one single mouse. Horizontal bars refer to median group values. **c-g** Data were obtained on day 9 post injection. ALT = alanine transaminase, CpG = 50μg or 100μg per mouse every other day, NI = not injected, PI = propidium iodide, PolyIC = 50μg or 200μg per mouse every other day, temp. = temperature, WT = wild-type. * *p* < 0.05; ** *p* < 0.01; *** *p* < 0.001; Mann-Whitney U test. Depicted data are from 1 experiment, representative of 2 experiments with 5 mice per experimental group

Since the statistical correlations did not provide conclusive evidence for a link between the extent of viral replication and the severity of the HLH-like symptoms in the mouse model, we investigated whether lytic viral replication was an essential requirement to elicit HLH disease or whether non-cytolytic immunostimulation would suffice to induce HLH pathology. MCMV is a dsDNA virus that predominantly triggers TLR9 [22], and therefore persistent CpG-mediated stimulation of TLR9 would be a valid way to mimic MCMV-mediated immune cell triggering. In fact, this strategy has been reported in C57BL/6 mice as a mouse model of secondary HLH or macrophage activation syndrome [23]. In addition to TLR9, TLR3 plays a significant, though less crucial role in the immune response against MCMV by recognizing dsRNA that is presumably formed during bidirectional transcription of the MCMV genome [22]. We therefore examined the effects of repetitive TLR9, TLR3 or combined TLR3 and -9 triggering on the induction of HLH-like pathology, in a dose-response set-up. As previously reported in C57BL/6 mice [23], repetitive CpG stimulation of BALB/c mice resulted in a mild HLH syndrome (Fig. 3). Weight loss occurred, but the mice recovered without fatalities (Fig. 3a). No hyper- or hypothermia was observed (Fig. 3b). The mice did develop hepatosplenomegaly, and lymphadenopathy only following the highest dosage of CpG (Fig. 3c). Hematologically, CpG-stimulated mice developed thrombocytopenia and anemia, though no lymphopenia (Fig. 3d). Plasma ferritin levels were elevated (Fig. 3e), but remained an order of magnitude lower than the levels measured in the MCMV-induced secondary HLH mouse model [13]. Splenic NK cells decreased significantly (Fig. 3f), while liver enzymes were not increased following chronic TLR9 activation (Fig. 3g). In contrast, repetitive stimulation of TLR3, using the TLR3 agonist Poly(I:C), was not able to elicit pronounced HLH-like symptoms in WT BALB/c mice. Almost no weight loss was observed (Fig. 3a) and the mice did not develop fever or hypothermia (Fig. 3b). Hepatosplenomegaly, lymphadenopathy, lymphopenia and anemia were absent (Fig. 3c, d), only thrombocytopenia occurred at the highest concentration of Poly(I:C) (Fig. 3d). Spleen NK cells were not decreased (Fig. 3f), but the highest dose of Poly(I:C) did elicit a slight increase in the plasma levels of ferritin and ALT, though not statistically significant (Fig. 3e, g). Thus, in contrast to repeated stimulation of TLR9, persistent triggering of TLR3 was not sufficient to induce HLH pathology in WT BALB/c mice. Lastly, the combined stimulation of TLR3 and TLR9 did not result in any additive pathological effects. The combinatory activation of both TLRs mostly mirrored the effects of TLR9 agonism alone, indicating that in contrast to TLR9, TLR3 activation is of minor importance in murine secondary HLH pathogenesis.

### Antibody-neutralized or UV-inactivated MCMV is no longer able to induce an HLH-like syndrome in WT BALB/c mice

**Fig. 4:**
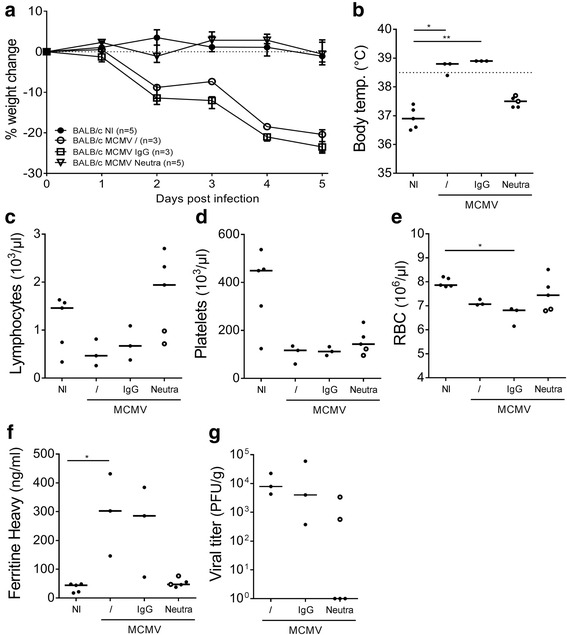
Neutralization of MCMV with polyclonal antibodies inhibits development of fulminant HLH in WT BALB/c mice. **a** Percentage change in body weight relative to initial body weight at day 0 post infection (p.i.). Median with interquartile range of 3-5 mice per experimental group. **b** Rectal body temperature (°C) on day 2 p.i. Dotted line = 38.5°C (fever). **c-e** Absolute lymphocyte, platelet and red blood cell (RBC) count in whole blood. **f** Plasma concentration of the ferritin heavy chain (ng/ml). Dots represent the average of two dilutions for one single mouse. Dotted line represent the lower ELISA detection limit. **g** Titer of infectious virus in spleen (PFU per g spleen tissue). **b-g** Data were obtained on day 5 p.i.. Dots represent individual animals. / = MCMV without prior incubation, IgG = MCMV incubated 1h with isotype control antibodies, Neutra = MCMV incubated 1h with anti-MCMV neutralizing antibodies as described in M&M, NI = not infected, RBC = red blood cells, temp. = temperature. ○ = open dots in the ‘Neutra’ group indicate mice in which neutralization was not completely efficient. * *p* < 0.05; ** *p* < 0.01; Kruskal-Wallis with Dunn’s post-test for multiple comparisons. Depicted data are from one experiment

Since a viral infection activates the host immune system in multiple intricate ways, solely TLR triggering is most likely insufficient to recapitulate the full response of the host to persistent MCMV infection. To establish whether challenges with viral particles in the absence of lytic viral replication would be sufficient to induce HLH disease, two different methodologies were used. In a first approach, MCMV was neutralized *in vitro* with MCMV-specific polyclonal antibodies [15], prior to infection of the mice. As an isotype control, MCMV treated with a cocktail of irrelevant polyclonal IgG antibodies was used. In contrast to isotype-treated MCMV, neutralized MCMV was no longer capable to induce full-blown HLH disease (Fig. 4). No weight loss, fever, lymphopenia, anemia, or hyperferritinemia developed (Fig. 4a-c, e, f). Only thrombocytopenia was observed (Fig. 4d). Interestingly, the neutralization was less efficient in 2 mice, that still displayed infectious virus in the spleen on day 5 post infection (Fig. 4g). These mice, indicated as open dots in Fig. 4, developed more pronounced HLH-like features than did their completely neutralized counterparts, corroborating a role for productive viral replication in the onset of murine virus-associated secondary HLH.

**Fig. 5:**
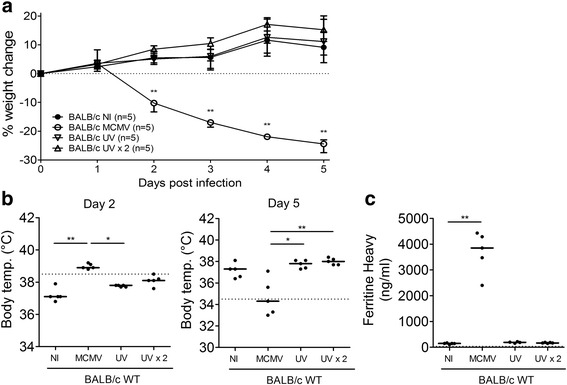
Infection of WT BALB/c mice with UV-inactivated MCMV does not elicit an HLH-like syndrome. UV = 5 x 10^3^ PFU of UV-inactivated MCMV, UV x 2 = 10^4^ PFU of UV-inactivated MCMV, administered every other day. **a** Percentage change in body weight relative to initial body weight at day 0 post infection (p.i.). Median with interquartile range of 5 mice per experimental group. **b** Rectal body temperature (°C) on day 2 (left panel) and on day 5 p.i. (right panel). Dotted line = 38.5°C (fever) or 34.5°C (endpoint as an indication of mortality). Dots represent individual animals. **c** Plasma concentration of the ferritin heavy chain (ng/ml) on day 5 p.i.. Dots represent the average of two dilutions for one single mouse. Dotted line represents the lower ELISA detection limit. **b-c** Horizontal bars refer to median group values. NI = not infected, PFU = plaque-forming units, temp. = temperature. * p < 0.05; ** p < 0.01; Kruskal-Wallis with Dunn’s post-test. Depicted data are from one experiment.

In the second approach, a sustained, non-replicating MCMV infection was mimicked by inactivating the virus through UV light exposure. The non-infectious viral particles were repeatedly administered to WT BALB/c mice to reproduce persistent MCMV-antigenic immunostimulation. UV-inactivated MCMV produces no direct lytic effects as it cannot replicate nor induce viral gene expression, but can still trigger pathogen receptors [24–26], allowing the study of MCMV-related immunostimulation without any confounding cytolytic effects. Repeated administration of inactivated virions did not induce clinical HLH disease in the mice. No weight loss (Fig 5a), pronounced fever (Fig. 5b, left panel), or hypothermia (Fig. 5b, right panel) developed. Additionally, ferritin plasma levels were not elevated (Fig. 5c). In conclusion, abortive infection with either antibody-neutralized or UV-inactivated MCMV was not sufficient to initiate HLH pathogenesis in WT BALB/c mice.

### Restriction of viral replication by cidofovir rescues MCMV-infected IFN-γ-KO mice from a lethal HLH-like syndrome

**Fig. 6:**
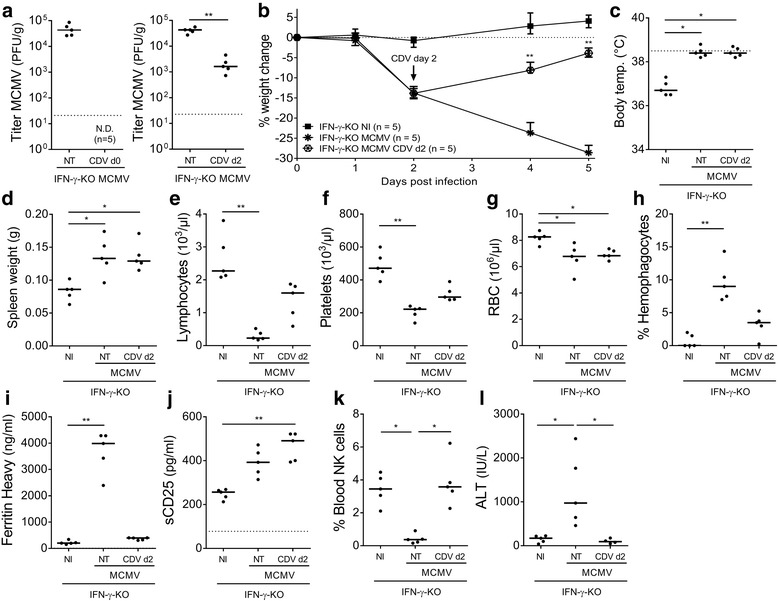
Antiviral cidofovir treatment rescues MCMV-infected IFN-γ-KO BALB/c mice from severe HLH-like disease. **a** Titer of infectious virus in spleen (PFU per g spleen tissue) with/without cidofovir (CDV) treatment on day 0 (d0) (left panel) or day 2 (d2) (right panel) post infection (p.i.). Plaque assay detection limit 2 PFU/organ, indicated by dotted line. **b** Percentage change in body weight relative to body weight at day 0 p.i.. Median with interquartile range of 5 mice per experimental group. Weight change was significantly different between infected CDV-treated and infected non-treated mice, on day 4 and 5 p.i. (**). Arrow indicates time point of CDV treatment. **c** Rectal body temperature (°C) on day 2 p.i.. Dotted line = 38.5°C (fever). **d** Absolute spleen weight (g). **e-g** Absolute lymphocyte, platelet and red blood cell (RBC) count in whole blood. **h** Percentage of hemophagocytes detected in cytospins of blood-derived WBC. **i** Plasma concentration of the ferritin heavy chain (ng/ml). **j** Plasma concentration of sCD25 (pg/ml). **k** Percentage of NK cells in whole blood, gated as CD122^+^CD49b^+^ cells of CD3^-^PI^-^ cells. **l** Plasma concentration of ALT (IU/L). **a, c-g, j-l** Dots represent individual animals. **h** Dots represent the average of triplicate counts of 100 cells from one individual mouse. **i** Dots represent the average of two dilutions for one single mouse. Horizontal bars refer to median group values. **i, j** Dotted lines represent the lower ELISA detection limit. **a, d-l** Data were obtained on day 5 p.i.. ALT = alanine transaminase, CDV = cidofovir 50mg/kg injected day 0 (d0) or day 2 (d2) p.i., NI = not infected, NT = not treated with CDV, PI = propidium iodide, temp. = temperature, WBC = white blood cells. * *p* < 0.05; ** *p* < 0.01; Mann-Whitney U test for single comparison, Kruskal-Wallis with Dunn’s post-test for multiple comparisons. Depicted data are from 1 experiment and representative of 2 independent experiments

To further investigate whether lytic viral replication was necessary to induce and drive progression of fulminant HLH disease, MCMV was administered to IFN-γ-KO mice together with a potent inhibitor of viral replication, the acyclic nucleoside phosphonate cidofovir. IFN-γ-KO mice were chosen in this setup since they display higher viral titers than WT BALB/c mice, presumably allowing for a more pronounced effect of the antiviral treatment. Cidofovir serves as an alternative substrate for the viral DNA polymerase, which upon incorporation into the viral DNA strand, obstructs further elongation [27]. In this way, cidofovir inhibits viral DNA synthesis but does not affect viral cell entry and expression of immunogenic immediate early and early antigens [28]. Thus, cidofovir prevents cellular damage due to lytic replication, but allows stimulation of extracellular and intracellular pathogen receptors by viral proteins. Cidofovir was administered at two different time points, to monitor the effects of MCMV on the initiation and further propagation of the HLH-like disease. Administration of cidofovir on day 0 p.i. efficiently inhibited viral replication, as no infectious virus could be detected in spleen tissue on day 5 p.i. (Fig. 6a, left panel). This prevented the development of the MCMV-induced HLH-like syndrome. Treated mice did not display weight loss or hypothermia, indicating 100% survival, whereas untreated infected IFN-γ-KO mice required euthanasia by day 5 p.i. according to institutional ethical policies. Key symptoms such as fever, lymphopenia, anemia, hemophagocytosis, lymphadenopathy, hyperferritinemia, elevated plasma levels of liver enzymes and decreased NK cell numbers were absent after cidofovir treatment (data not shown). Only splenomegaly and thrombocytopenia did develop in MCMV-infected IFN-γ-KO mice treated with cidofovir on day 0 (data not shown), suggesting that these symptoms may not be linked to viral replication but are rather consequent to the immune response elicited against immediate early and/or early MCMV antigens.

The potent effect of cidofovir on day 0 led us to investigate its therapeutic potential in limiting the further progression of HLH disease in the mouse model. As no drug-related effects of cidofovir were observed in naive IFN-γ-KO mice in the previous experiment, the treatment was considered safe and this experimental group was not included in the following experiments. Cidofovir was administered to MCMV-infected IFN-γ-KO mice on day 2 p.i., when first disease symptoms appeared. The treatment resulted in strongly reduced (20- to 70-fold) but detectable viral titers in spleen on day 5 p.i. (Fig. 6a, right panel). Weight loss quickly reversed after cidofovir treatment (Fig. 6b) and all cidofovir-treated mice survived the infection. The development of fever was unaltered as the treatment was only started after its onset (Fig. 6c). Many HLH-like symptoms were resolved or attenuated. Levels of ferritin, ALT, and the number of NK cells in blood were normalized (Fig. 6i, k, l), while lymphopenia, thrombocytopenia and hemophagocytosis were attenuated after cidofovir treatment (Fig. 6e, f, h). Nevertheless, splenomegaly, anemia and elevated plasma levels of sCD25 were present to a similar extent with or without therapeutic cidofovir treatment (Fig. 6d, g, j), indicating that these symptoms are less related to viral replication but probably represent the effects of immunopathologic mechanisms.

In conclusion, inhibition of lytic viral replication was able to prevent and cure the MCMV-induced HLH-like disease in IFN-γ-deficient mice, suggesting that both the initiation and propagation of full-blown HLH in this mouse model is dependent on viral replication and sustained viremia, even though a limited set of symptoms appears to be driven by immunopathology.

### Immunosuppressive dexamethasone treatment attenuates limited HLH symptoms in MCMV-infected WT BALB/c mice

The experiments with cidofovir suggested that at least part of the HLH-like syndrome was less dependent on viral replication but rather resulted from the concomitant immune response. CD8^+^ T cells and CD11b^+^ cells are highly activated post MCMV infection, as previously described [13], and the excessive immune activation may play a role in mediating immunopathology. To investigate this, an immunosuppressive drug was administered daily to MCMV-infected BALB/c mice starting day 2 post infection. Dexamethasone (DEX) was chosen as it is the preferred steroid treatment in the HLH-2004 therapeutic protocol [29].

**Fig. 7:**
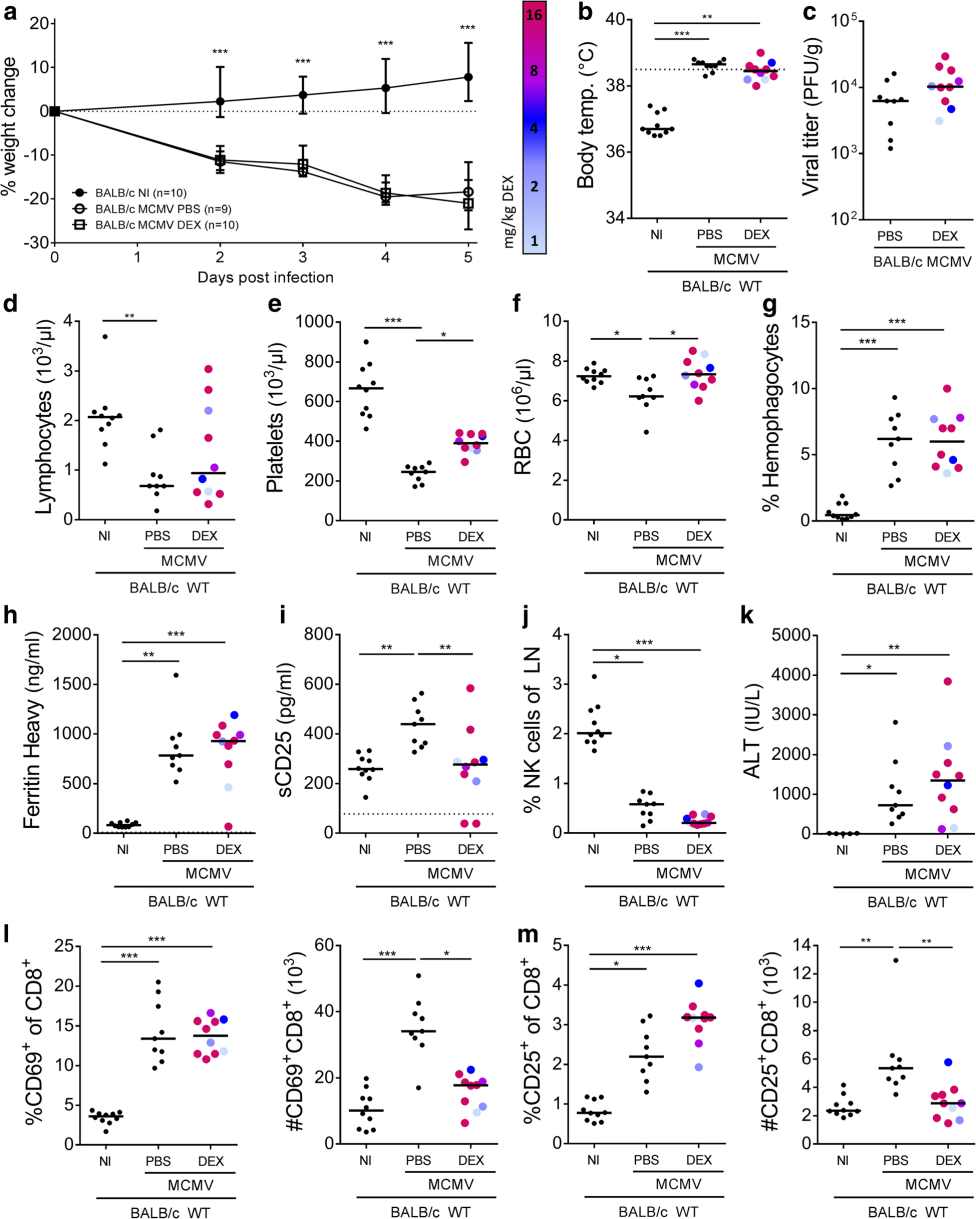
Daily dexamethasone treatment does not alleviate the HLH-like syndrome in MCMV-infected WT BALB/c mice. DEX = dexamethasone, range 1-16 mg/kg per day, starting from day 2 post infection (p.i.), indicated by color codes from blue to red for the lowest to highest dose. **a** Percentage change in body weight relative to initial body weight at day 0 p.i.. Median with interquartile range of 9-10 mice per experimental group. **b** Rectal body temperature (°C) on day 2 p.i.. Dotted line = 38.5°C (fever). **c** Titer of infectious virus in spleen (PFU per g spleen tissue). Plaque assay detection limit 2 PFU/organ. **d-f** Absolute lymphocyte, platelet and red blood cell (RBC) count in whole blood. **g** Percentage of hemophagocytes detected in cytospins of blood-derived WBC. **h** Plasma concentration of the ferritin heavy chain (ng/ml). **i** Plasma concentration of sCD25 (pg/ml). **j** Percentage of NK cells in lymph nodes (LN), gated as CD122^+^ CD49b^+^ cells of CD3^-^ ZombieAqua^-^ cells. Cells from both inguinal LN. **k** Plasma concentration of ALT (IU/L). **l, m** Percentage (left panel) and absolute number (right panel) of activated cytotoxic T cells from both inguinal lymph nodes, gated as CD69^+^ or CD25^+^ cells of CD8^+^ ZombieAqua^-^ cells. **b-f, i-m** Dots represent individual animals. **g** Dots represent the average of triplicate counts of 100 cells from one individual mouse. **h** Dots represent the average of two dilutions for one single mouse. Horizontal bars refer to median group values. **h, i** Dotted lines represent the lower ELISA detection limit. **c-m** Data were obtained on day 5 p.i.. ALT = alanine transaminase, NI = not injected, PFU = plaque-forming units, sCD25 = soluble CD25, temp. = temperature, WBC = white blood cells, WT = wild-type. * *p* < 0.05; ** *p* < 0.01; *** *p* < 0.001; Mann-Whitney U test for single comparison, Kruskal-Wallis with Dunn’s post-test for multiple comparisons. Depicted data are from two independent experiments

Weight loss post infection was not counteracted by DEX treatment (Fig. 7a) and the development of fever was unaltered since immunosuppression was only started after the onset of this symptom (Fig. 7b). Lymphopenia was still present, although the lymphocytes in some DEX-treated mice returned to normal levels (Fig. 7d). The drop in platelet numbers following MCMV infection was significantly attenuated in the animals receiving DEX (Fig. 7e). Hemophagocytosis, hyperferritinemia and increased levels of plasma liver enzymes were present to a similar extent in PBS-treated and DEX-treated MCMV-infected mice (Fig. 7g, h, k). Steroid treatment reduced the number of NK cells post infection even further (Fig. 7j). The most pronounced therapeutic effects of the immunosuppressive treatment were observed in the correction of anemia (Fig. 7f) and in the normalization of the plasma concentration of sCD25 (Fig. 7i), a measure for the degree of T cell activation in HLH. In line with the sCD25 data, the total number of activated CD8^+^ T cells in lymph nodes decreased following DEX treatment, although the relative percentage of activated cells in the CD8^+^ T cell population remained equal or even slightly increased (Fig. 7l, m). Notwithstanding the adequate suppression of T cell activation, the immunosuppressive treatment did not significantly hamper control over the viral infection, as demonstrated by similar splenic viral titers in the presence or absence of DEX (Fig. 7c).

In conclusion, immunosuppression using DEX was insufficient to temper the HLH-like syndrome in MCMV-infected WT BALB/c mice, although a limited number of parameters, i.e. the degree of anemia, thrombocytopenia and elevation of sCD25 levels, appeared to be immune-mediated.

## Discussion

Hemophagocytic lymphohistiocytosis is a hyperinflammatory disorder provoking high morbidity and risk of mortality in both children and adults. In recent cohorts, overall fatality has been estimated at 30%, but may vary considerably between different HLH subtypes [30]. In particular, HLH associated with active EBV disease is known for its aggressive disease course and poor prognosis [31]. As of yet, no specific treatments exist for virus-associated secondary HLH, and emphasis is placed on the rapid elimination of the triggering infection through antiviral therapy, combined with the standard immunosuppressive treatment [29]. To improve the outcome of virus-associated HLH and to devise more targeted therapies for this HLH subtype, its pathogenesis needs to be elucidated into further detail. In this manuscript, a recently described MCMV-induced mouse model of herpesvirus-associated secondary HLH was utilized to gain further insights into the role of viruses and the antiviral immune response in the development of HLH. Previous findings in this model had indicated that mice lacking the pro-inflammatory cytokine IFN-γ displayed a more severe spectrum of HLH, in comparison to their WT counterparts [13], suggestive of a protective role for the cytokine. These observations were highly relevant as they reflected the occurrence of IFN-γ-independent HLH-like disease in a number of interesting case reports (reviewed in references [32] and [33]), but were in clear contrast with data from primary HLH models, in which IFN-γ was designated as the key pathogenic cytokine since its depletion resulted in complete inhibition of the fatal HLH-like syndrome [8, 34]. The lack of antiviral IFN-γ activity and the correspondingly high viral titers detected in infected IFN-γ-deficient mice, led us to speculate a role for uncontrolled MCMV replication in the worsening of HLH-like disease. However, when WT mice were inoculated with doses of MCMV that were 2, 5, or even 25 times higher, mimicking unrestrained viral proliferation in the absence of IFN-γ, a similar disease spectrum as observed in IFN-γ-KO mice could not be induced [13]. Hence, other, non-virus-mediated mechanisms must play a role in disease development. The present article investigated into closer detail the respective contributions of virus-mediated pathology and immunopathology in the development of secondary HLH.

Comparison of splenic viral titers in WT C57BL/6, WT BALB/c and IFN-γ-KO mice post infection with MCMV revealed a trend of increasing viral load in the mouse strains that developed HLH. Resistant C57BL/6 mice managed to clear the virus and displayed low to undetectable titers of MCMV, while IFN-γ-KO mice, who develop the most complete spectrum of HLH, presented with the highest viral titers, consistent with the hypothesis that the severity of the HLH-like syndrome in the mutant mice was directly linked to increased lytic viral replication. Nonetheless, the splenic viral titer of infected WT and IFN-γ-KO BALB/c mice differed on average only 5-fold, while both strains displayed very different HLH symptoms [13], indicating that increased disease severity may not be exclusively attributed toaugmented viral proliferation but may emanate from additional differences in immune activation and immunopathology. Indeed, correlation analysis of the splenic viral titer in infected mice with key HLH symptoms such as fever, cytopenia, hyperferritinemia, etc. supported no direct relationship between the extent of viral replication and the severity of murine HLH. On the other hand, repeated immunostimulation with UV-inactivated MCMV particles did not induce any HLH-like symptoms in WT BALB/c mice, indicating that the HLH syndrome is -at least for its induction- dependent on a replication-competent viral agent. Neutralization of MCMV with polyclonal antibodies confirmed these findings, as the development of full-blown HLH was completely abolished in neutralized MCMV-injected mice. Similar results were obtained when the replication of MCMV was halted *in vivo*, by administering the potent antiviral agent cidofovir at the time point of infection. In the absence of viral replication, the general HLH syndrome was not observed, although individual features like splenomegaly and thrombocytopenia did develop. Cidofovir treatment was also used to halt viral replication *in vivo* at a later time point of the infection, in order to investigate whether ongoing viral replication and sustained viremia were necessary to propagate HLH. The antiviral treatment attenuated the HLH-like syndrome, reversing weight loss and rescuing all infected mice. Analogous to the earlier cidofovir treatment, some HLH-like symptoms were not reduced by late treatment with cidofovir, i.e. splenomegaly, anemia and elevated sCD25 were still present, while lymphopenia, thrombocytopenia and hemophagocytosis were attenuated but not absent. Disease initiation and progression to full-blown HLH is thus dependent on viral replication, although a limited number of symptoms may be mediated by the immune response against viral proteins, such as the immediate early and early MCMV antigens.

Neutralization of MCMV with polyclonal antibodies not only allowed to focus on the role of viral infection in the HLH mouse model, but also permitted to exclude a pathogenic role for any other contaminating components in the MCMV stock solution, derived from salivary gland (SG) homogenates. In literature, there has been some debate regarding the use of SG-derived viral preparations, as they contain a crude mixture of not only viral particles but also cytokines, chemokines, TLR ligands, hormones, and other cellular proteins. As these components originate from inflamed, immunologically active tissue [20, 35], they could contribute to the inflammatory reaction following infection with SG-MCMV. *In vivo* infection with SG-MCMV is known to be more aggressive than cell culture-passaged MCMV. The difference in virulence can on the one hand be explained by the presence of immuno-active components in SG homogenates, but may on the other hand be related to a partial loss in host affinity or change in organ or cellular tropism following serial passages *in vitro* [18, 21, 36–38]. Tissue-derived components in SG-MCMV may increase the inflammatory reaction to viral inoculation and could speed up the development of immunopathology, as such being co-responsible for provoking HLH in the murine model. Nonetheless, injection with neutralized SG-MCMV pointed out that the non-viral immuno-inflammatory components present in SG homogenates were not able to elicit HLH-like disease by themselves, indicating that the uncontrolled viral infection was indeed the key driver of full-blown HLH development. Synergistic activities between the virus and non-viral components can however not be excluded from these experiments.

Uncontrolled pathogen replication has been emphasized as a pathogenic factor in other animal models of infection-associated secondary HLH. In an EBV-induced humanized mouse model, mice displayed persistently high viremia and disease severity correlated with EBV DNA production. Plasma EBV titers also correlated with the level of T cell hyperactivation and IFN-γ hypersecretion, linking the magnitude of the productive infection to the development of immunopathology. Additionally, inoculation of the humanized mice with heat-inactivated EBV did not induce any HLH symptoms [39], similar to our results with UV-inactivated MCMV. Likewise, in a Salmonella-induced mouse model of bacteria-associated HLH, disease severity was correlated with high splenic and hepatic bacterial loads. Only the most severely infected mice fulfilled six of the eight HLH-2004 diagnostic criteria. The bacterial load could statistically be linked to the severity of individual HLH symptoms such as cytopenia, hepatosplenomegaly and hyperferritinemia [40]. In the MCMV-induced mouse model, however, we could not confirm a direct statistical relationship.

Models of primary HLH are equally reliant on sustained viremia and ongoing viral replication to induce the HLH syndrome [8, 9]. Due to the inherent defect in granule-mediated cytotoxicity, the viral trigger, in most cases lymphocytic choriomeningitis virus (LCMV), cannot be cleared and proliferation is unrestrained. Nonetheless, the extent of viral replication does not seem to correlate fully with disease severity. In LCMV-infected syntaxin11-deficient mice, the HLH syndrome progressed from severe, acute disease to a chronic and moderate syndrome, irrespective of the viral titer [10]. Furthermore, cytokine hyperproduction and CD8^+^ T cell activation in LCMV-infected perforin- and Lyst-deficient mice were independent of the splenic viral load [41]. Antibodies against LCMV, neutralizing the serum viral titer in perforin-deficient mice, were shown to mitigate the disease, allowing for prolonged survival in a part of the mice, but not full recovery. On the other hand, chronic administration of a synthetic LCMV peptide, simulating persistent antigen stimulation, only partially replicated the HLH syndrome in perforin-deficient mice, in a milder degree as well [8]. Together, these data indicate a partial dependence of the primary HLH model on unrestricted LCMV replication.

In line with the findings in primary and secondary HLH mouse models, reports of patients with virus-associated HLH corroborate the involvement of sustained viremia in the severity and outcome of the syndrome. In adult secondary HLH, the presence of a viral infection constituted a prognostic factor for poor outcome [42]. In EBV-associated HLH, EBV genome copy numbers were correlated with disease outcome. Patients who achieved remission managed to control the virus after treatment initiation, while patients who did not respond to therapy continued to present with high EBV copy numbers in serum [43]. The viral load may also be used to distinguish EBV-associated HLH from the less severe EBV-induced infectious mononucleosis. Viral titers in patients with EBV-associated HLH can be up to 10^4^ times higher when compared to patients with infectious mononucleosis [43, 44]. In addition, the percentage of EBV-infected peripheral blood mononuclear cells is on average 10 times higher in EBV-HLH, compared to infectious mononucleosis [45].

To further dissect the contributions of virus- versus immune-mediated pathology in the pathogenesis of MCMV-induced murine HLH, we investigated whether immunopathology mediated by excessive triggering of pathogen receptors played a role. Virus-mediated immunostimulation was mimicked by administering TLR ligands to the mice. Repeated administration of Poly(I:C), a TLR3 ligand, did not induce any HLH-like symptoms, while repetitive injections of CpG, a TLR9 agonist, did. These results are in line with observations made by Behrens et al. [23], who reported a failure to induce HLH-like disease in C57BL/6 mice repeatedly injected with Poly(I:C). Instead, chronic stimulation of TLR9 with unmethylated CpG oligonucleotides was found to be unique in triggering the syndrome. Interestingly, dsDNA viruses, like EBV, HCMV, MCMV and other herpesviruses, notoriously linked to active HLH episodes [46], activate TLR9 signaling during acute infection. Excessive TLR9 triggering has also been linked to the development of HLH in a second mouse model [47]. In another model of secondary HLH, using IL-6 transgenic mice, an exaggerated response to TLR stimulation with Poly(I:C) as well as with CpG was observed, but no HLH-like syndrome developed. Solely injection with lipopolysaccharide (LPS), a TLR4 ligand, induced an acute HLH phenotype in the IL-6 transgenic mice, possibly mirroring the syndrome of bacteria-induced HLH [48].

The role of immune-mediated pathology was further examined by treating MCMV-infected BALB/c mice with an immunosuppressant. The steroid dexamethasone was chosen, considering its preferred use in the HLH-2004 therapeutic guidelines [29]. Although dexamethasone was not able to cure the mice, it did reduce the extent of anemia and thrombocytopenia post infection and inhibited the increase in plasma sCD25. Combined with the findings from the cidofovir experiments, it appears that the development of cytopenias and elevated sCD25 levels are mostly immune-mediated. Indeed, it is known that during systemic inflammatory reactions, i.e. following viral infection, LPS administration or injection with proinflammatory cytokines, platelets are attracted to the liver and/or lungs, where they accumulate, decreasing their numbers in peripheral blood and resulting in thrombocytopenia [49, 50]. Liver platelet accumulation has also been described in response to replication-deficient, non-cytolytic adenoviruses, demonstrating the immunological nature of the process [51]. Likely, anemia also results from excessive immune activation and the cytokine storm. Many HLH-related and MCMV-induced cytokines are known to suppress hematopoiesis, amongst which IL-1β, IFN-γ and TNF-α [52], whereas IL-6 has the potency to specifically induce anemia [53]. However, as the anemia developed acutely post infection, decreased erythropoiesis is probably not the main cause, but rather increased consumption and destruction of erythrocytes in the inflammatory response [54], a process termed “consumptive anemia of inflammation” [55]. Lastly, plasma sCD25 has been shown to be shed by hyperactivated CD8^+^ T cells [56] and is thought to reflect the degree of T cell activation in HLH, suggesting an immunological background for this symptom as well.

Immunosuppressive treatment has been examined in other animal models of HLH. In a humanized, transgenic mouse model of secondary HLH, treatment with dexamethasone or intravenous immunoglobulins did not succeed to save the mice from fatal HLH [57], in line with our findings using dexamethasone. In the LCMV-infected perforin-deficient mouse model, dexamethasone therapy was not successful either, nor was chemotherapeutic treatment with cisplatin, clofarabine, doxorubicin, fludarabine, 5-fluorouracil or vinblastine [58]. Only etoposide, cyclophosphamide and methotrexate, all agents proposed in the HLH-2004 therapeutic protocol, substantially alleviated symptoms and allowed survival of the LCMV-infected perforin-deficient mice [58]. Thus, although dexamethasone was inadequate in the MCMV-induced mouse model, other immunosuppressive agents may have a more potent effect and may further unravel the role of immunopathology in virus-associated secondary HLH.

## Conclusions

Our data demonstrate that infectious viral titers do not correlate with the severity of key HLH features, although the titer is the highest in mouse strains developing an HLH-like syndrome post MCMV infection. Lytic viral replication and sustained viremia played an essential part in the pathogenesis since abortive viral infection and extracellular stimulation of pathogen receptors by MCMV-specific antigens was insufficient to induce a full-blown HLH-like syndrome. Nonetheless, a limited set of symptoms, in particular anemia, thrombocytopenia and elevated soluble CD25, appeared less dependent of the viral replication but rather immune-mediated, as corroborated by immunosuppressive treatment of infected mice with dexamethasone. Thus, distinguishing the pathogenic effects of the virus itself from the immunological consequences of viremia is not straightforward. Most likely, a certain level of viremia is necessary to elicit the aberrant immune responses driving HLH pathogenesis. Therefore, in clinical practice, it remains equally important to halt the rampant immune system and to eliminate persisting infections by applying a combined treatment of immunochemotherapy and supportive pathogen-directed therapy. Case reports of patients with virus-associated HLH confirm that monotherapy with antivirals is usually insufficient to cure full-blown HLH [3, 6, 59].

## Abbreviations

ALT: Alanine transaminase
ATCC: American Type Culture Collection
CDV: Cidofovir
DAMPs: Danger-associated molecular patterns
DEX: Dexamethasone
dsDNA: Double-stranded DNA
EBV: Epstein-Barr virus
H&E: Hematoxylin and eosin staining
HCMV: Human cytomegalovirus
HLH: Hemophagocytic lymphohistiocytosis
i.p.: Intraperitoneal
IFN: Interferon
KO: Knockout
LCMV: Lymphocytic choriomeningitis virus
LPS: Lipopolysaccharide
MCMV: Mouse cytomegalovirus
NH_4_Cl: Ammonium chloride
NI: Not infected
NK cell: Natural killer cell
p.i.: Post infection
PBS: Phosphate-buffered saline
PFU: Plaque-forming unit
PI: Propidium iodide
Poly(I:C): Polyinosinic-polycytidylic acid
RBC: Red blood cells
sCD25: Soluble CD25
SG: Salivary gland
TLR: Toll-like receptor
WBC: White blood cells
WT: Wild-type
